# Prevention of stillbirths associated with umbilical cord abnormalities: A clinico‐pathological overview

**DOI:** 10.1002/ijgo.70639

**Published:** 2025-11-20

**Authors:** Laura Avagliano, Chiara Parodi, Valentina Massa, Gaetano Bulfamante

**Affiliations:** ^1^ University of Milan Milan Italy; ^2^ Department of Health Sciences University of Milan Milan Italy; ^3^ Department of Biomedical, Surgical and Dental Sciences University of Milan Milan Italy; ^4^ Toma Advanced Biomedical Assays S.p.A. Varese Italy

**Keywords:** knot, nuchal cord, placenta, pregnancy, stillbirth, stricture, ultrasound, umbilical cord, velamentous insertion

## Abstract

Stillbirths affect millions of pregnancies every year worldwide, and obstruction of the umbilical cord blood flow is one of the main causes of fetal death. This review provides a clinico‐pathological overview of cord abnormalities potentially associated with antepartum stillbirth, describing the mechanism determining the death and addressing suggestions about the possibility of stillbirth prevention. Fatal effects on the fetal circulatory system can occur due to various umbilical cord abnormalities, both in cases of acquired anomalies (i.e., multiple tight nuchal cords and true knots) or congenital anomalies (i.e., aneurysmatic malformation, stricture, abnormalities of cord insertion in the fetal or placenta site such as velamentous or furcate cord insertion). In pregnancies with an identification of umbilical cord abnormality, ultrasound signs of fetal well‐being could be monitored over time. Although close monitoring cannot prevent a sudden lethal obstruction of umbilical cord vessels, it could identify the development of long‐lasting signs of fetal decompensation, possibly preventing fetal death.

## 1 INTRODUCTION

Stillbirth remains a major global health burden, affecting millions of pregnancies each year worldwide (United Nations Inter‐Agency Group for Child Mortality Estimation [UN IGME], 2023).^1^ Among the various etiologies, impairment of umbilical cord blood flow represents a leading cause of fetal death.^2^, ^3^, ^4^ Umbilical cord abnormalities have been implicated in a substantial proportion of stillbirths, although reported prevalence varies widely, from 3% to 10% to over 60%.^5^, ^6^, ^7^ Such variability largely reflects a knowledge gap, and it is related to differences in classification systems, diagnostic approaches, and inclusion criteria across studies.^5^

The aim of this narrative review is to provide a clinico‐pathological overview of cord abnormalities associated with antepartum stillbirth in singleton pregnancies, highlighting potential underlying mechanisms. The objective is to provide information on potential strategies that could contribute to the prevention of stillbirth.

## 2 METHODS OF THE NARRATIVE REVIEW

### 2.1 Literature search strategy

The writing and reporting of this narrative review were guided by the SANRA (Scale for the Assessment of Narrative Review Articles) criteria. We included in our review studies with clinico‐pathological data (both original articles or case reports), guidelines, and meta‐analyses, reporting results about umbilical cord characteristics and stillbirth. The PubMed databases were systematically searched, considering papers published in English from the inception of the database through to July 2025. Titles and abstracts were collected and screened independently by two authors (LA, CP). After removal of duplicates, full‐text versions of all selected publications were examined.

### 2.2 Image source and ethical compliance

All pathological and clinical images presented in this manuscript originate from the personal archives of two of the authors (LA, GB) and are reproduced with parental consent. The images have been fully anonymized and are used exclusively for scientific and educational dissemination.

## 3 UMBILICAL CORD ABNORMALITIES POTENTIALLY ASSOCIATED WITH PREVENTABLE STILLBIRTH

### 3.1 Nuchal cord

#### 3.1.1 Definition and prevalence

The presence of a complete loop of the umbilical cord around the whole circumference of the fetal neck is referred to as a nuchal cord. The nuchal cord is a dynamic condition, able to resolve spontaneously during the intrauterine life.^8^ It is relatively frequently observed, and the widespread application of new technologies has provided high accuracy in its prenatal ultrasound detection, with a sensitivity up to 96.7% and specificity up to 96%.^8^ A recent meta‐analysis did not observe an association between nuchal cord and stillbirth.^5^ However, multiple and/or tight nuchal cords are less likely to spontaneously resolve during pregnancy, increasing the risk of stillbirth,^3^ with an estimated odds ratio of up to 13.40 (95% confidence interval [CI] 1.12–160.34) in the presence of four loops.^9^ In a cohort study, multiple nuchal cords have been observed in 3.7% of stillbirths.^10^

#### 3.1.2 Mechanism of death

The causal link between nuchal cord and stillbirth remains poorly investigated. However, the mechanism that causes the death could be related to the interruption of umbilical blood flow by an obstructive mechanism of compression and/or traction of the umbilical vessel.^11^ While some cases of death occur as acute events, others might develop from subacute or chronic mechanisms. A tight nuchal cord can cause an obstruction of jugular venous return. This obstruction can lead to congestion of cerebral and meningeal vessels, causing intracranial hemorrhage and leading to death.^12^ Moreover, compression of the carotid arteries by umbilical cord loops can raise blood pressure, redistributing fetal blood to vital organs like the brain and heart.^13^, ^14^ This, together with the carotid chemoreflex, can increase blood flow through the ductus venosus and the umbilical vein, leading to cardiac remodeling^13^, ^14^ and increasing the risk of fetal death.

#### 3.1.3 Relevant diagnostic findings

**FIGURE 1:**
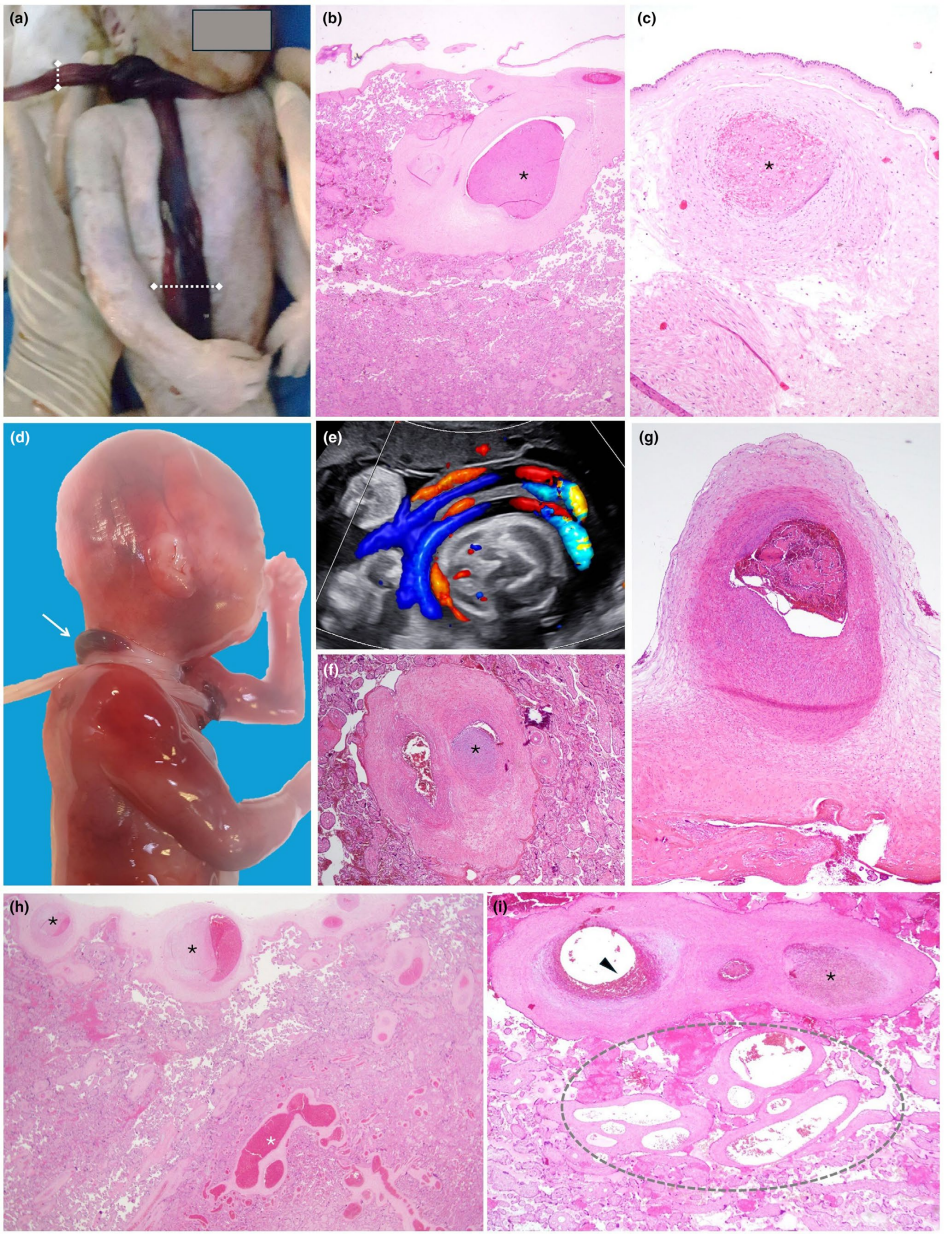
Nuchal cord: Macroscopic and histological key findings supporting its role in stillbirth. (a) Stillborn baby affected by a tight nuchal cord. Note the difference in umbilical cord diameter before and after the obstruction (dotted lines). (b) Chorionic vessel with early occlusive firmly attached thrombus (asterisk) (staining: Hematoxylin and eosin [H&E]; original magnification ×4). (c) Old occlusive thrombus of a chorionic vessel (asterisk) (H&E; ×4). Histology confirms that previous cord obstruction events might leave lasting vascular changes, providing evidence for cumulative fetal risk. (d) Stillborn baby affected by multiple nuchal cords. Note the difference in color of the umbilical cord before and after the site of obstruction (arrow). (e) Two‐dimensional ultrasound using color Doppler showing multiple cord loops around the neck in a transverse scan. (f) Large endothelial cushion in stem vessel (asterisk) (H&E; ×10); clinically, this might suggest gradual fetal blood flow compromise. (g) Chorion vessel with recent thrombosis in a vessel affected by an old cushion (H&E; ×4), clinically providing evidence for cumulative events of blood flow impairment. (h) Placental effects of nuchal cord: Section of the upper third of the placenta showing multiple sites of intramural fibrin deposition (endothelial cushion) on chorionic vessels (black asterisks) and vascular ectasia (greater than fourfold difference in diameter of the adjacent vessels) of stem villous vessels (white asterisk) (H&E; ×4). These histological changes correlate with impaired fetal circulation. (i) Placental effects of nuchal cord: Recent (arrowhead) subocclusive and old (asterisk) occlusive thrombosis of stem vessels. Note also the vascular ectasia (dotted circle) (H&E; ×10). The same histological lesions can affect cases with other types of blood flow occlusion, such as true knots.

In stillbirths where umbilical vessel occlusion occurs intermittently before death, the prolonged process can produce distinct gross and histological signs in the cord and placenta, varying by location: areas distal to the obstruction show interrupted blood flow, while proximal areas display vascular congestion and increased pressure. Gross examination of the cord might reveal changes in diameter and coloration (Figure 1). The placenta often shows abnormalities in blood circulation, including histological lesions in chorionic vessels near the cord insertion^15^ and along the villous tree. The increased pressure in the chorionic plate and stem villous veins can lead to fibrin accumulation within the vessel wall, forming intimal fibrinoid endothelial cushions^11^ (Figure 1).

**FIGURE 2:**
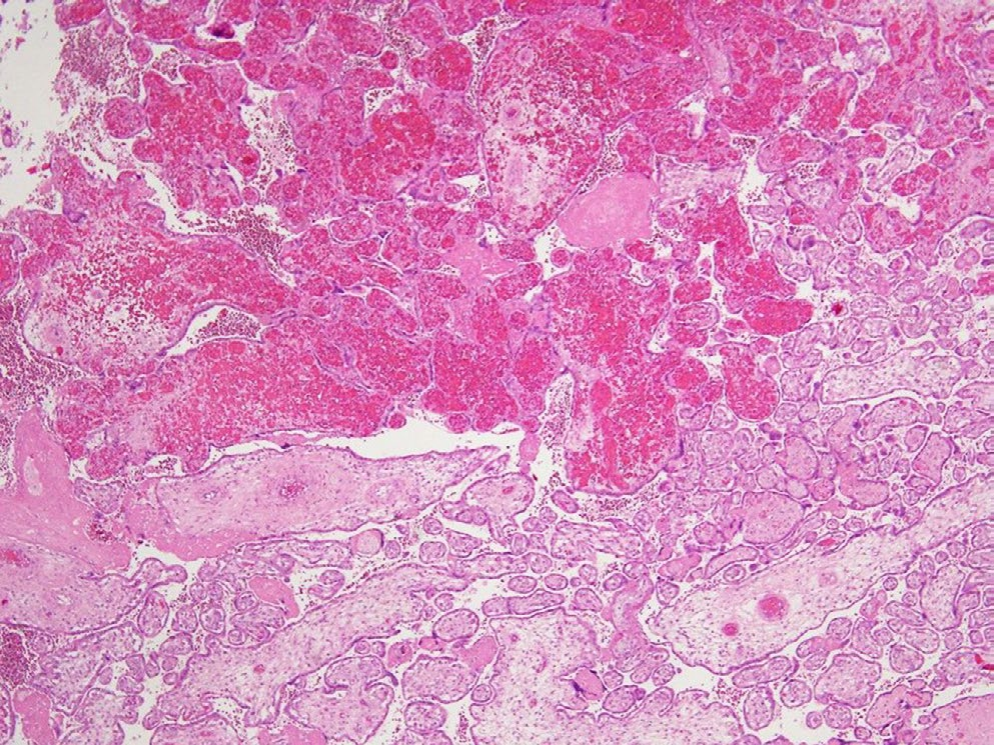
Intravillous hemorrhage: a potential sign of abrupt changes in fetal capillary pressure. Acute intravillous hemorrhage with extravasated but largely intact fetal red blood cells spread into the villous stroma, as a result of conditions that cause abrupt changes in fetal capillary pressure (H&E; ×20).

The accumulation of fibrinoid matrix can appear as a recent, early cushion of intramural fibrin deposition or, in cases of recurrent, non‐immediately fatal events, it can become mineralized (remote, old intramural fibrin deposition) (Figure 1).^11^ Increased venous pressure can also lead to vascular ectasia. In this condition, a localized luminal dilation is observed: the diameter of vessels in the chorionic plate or stem villi is at least four times increased compared to the luminal diameter of the neighbor vessels (Figure 1).^11^ Intravillous hemorrhage (Figure 2) polarized toward the chorionic plate can be another sign of abnormal fetal blood pressure: it can be a result of back pressure induced by acute umbilical or chorionic venous obstruction.^16^ Villous edema can also be observed in stillbirth with acute fetal blood flow obstruction. In this condition, the edema in the villous tree reflects the fluid balance dysregulation related to the obstruction.^16^, ^17^

Fetal autopsy can show cardiac modifications and heart remodeling, aiming to discriminate between a sudden death versus a long‐lasting process.

#### 3.1.4 Possible strategies for prevention or surveillance

Regarding the possibility of stillbirth prevention, the scientific community agrees that loose loops of the nuchal cord and a single nuchal cord are generally associated with normal fetal–neonatal outcomes^5^, ^18^ and therefore no intervention is needed. To date, obstetric and gynecological professional societies (e.g., American College of Obstetricians and Gynecologists, Royal College of Obstetricians and Gynecologists) have not issued recommendations supporting early delivery in pregnancies with an incidentally detected single nuchal cord, reflecting the general consensus that this finding is usually benign. Conversely, the clinical management of multiple nuchal cords remains challenging. Some authors have suggested case‐specific management with increased antepartum fetal surveillance^19^ and consideration of early‐term delivery beyond 37 weeks of gestation with the aim of preventing the risk of stillbirth during the final weeks of pregnancy.^8^

Emerging evidence on assessing fetal cardiac remodeling,^13^, ^14^ particularly changes in ductus venosus or umbilical venous flow, might also offer a potential tool for early detection of hemodynamic compromise and could be included in preventive monitoring protocols.

### 3.2 True knot

#### 3.2.1 Definition and prevalence

A true knot forms when the fetus passes through a cord loop, creating an entwining of the cord around itself. It affects 0.26% to 1% of births,^5^, ^9^, ^12^ with a reported range of occurrence from 0.3% to 2.1% of pregnancies.^20^

A true knot is not inherently problematic, as Wharton's jelly usually protects the vessels. However, thin cords are more susceptible to occlusion. Adverse outcomes arise from increasing vascular compression as the true knot tightens, leading to further vascular constriction and, potentially, complete blood flow occlusion. While loose true knots might be tolerated, tight true knots significantly raise stillbirth risk compared with pregnancies without true knots (odds ratio [OR] 3.96, 95% CI 1.85–8.47,^5^ up to an OR 15.46, 95% CI 9.30–25.70^9^).

#### 3.2.2 Mechanism of death

The mechanism that causes the death is like those described for the nuchal cord, and it is related to the interruption of fetal blood flow in the umbilical vessels.

#### 3.2.3 Relevant diagnostic findings

**FIGURE 3:**
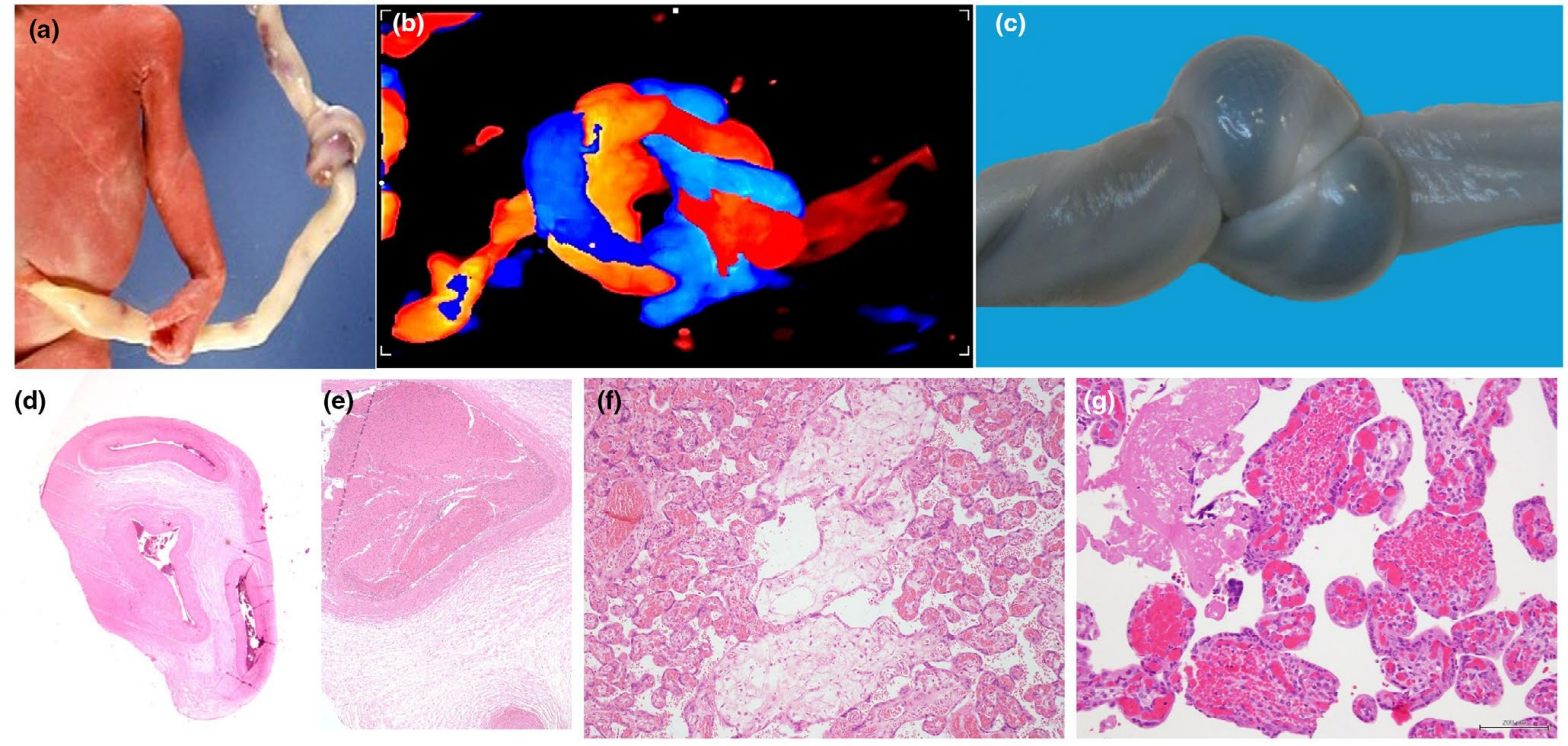
Umbilical cord true knot: macroscopic and histological key findings supporting its role in stillbirth. (a) Stillborn baby affected by umbilical cord true knot. Note the difference in the color of the umbilical cord before and after the knot, as a sign of blood flow interruption. (b) Color Doppler ultrasound appearance of the umbilical cord true knot. (c) Macroscopic aspect of the umbilical cord true knot. (d) Collapse of all the vessels of the umbilical cord in the segment of the umbilical cord located between the knot (H&E; ×2), as a sign of mechanical compression of the vessel. (e) Thrombosis of the umbilical vein in the segment of the umbilical cord located between the knot and the placenta (H&E; ×10). (f) Severe villous edema (in the center of the image), reflecting acute obstruction of blood flow from the placenta to the fetus (H&E; ×10). (g) Intravillous hemorrhage, reflecting an acute obstruction of peripheral fetal venous blood flow (H&E; ×20).

**FIGURE 4:**
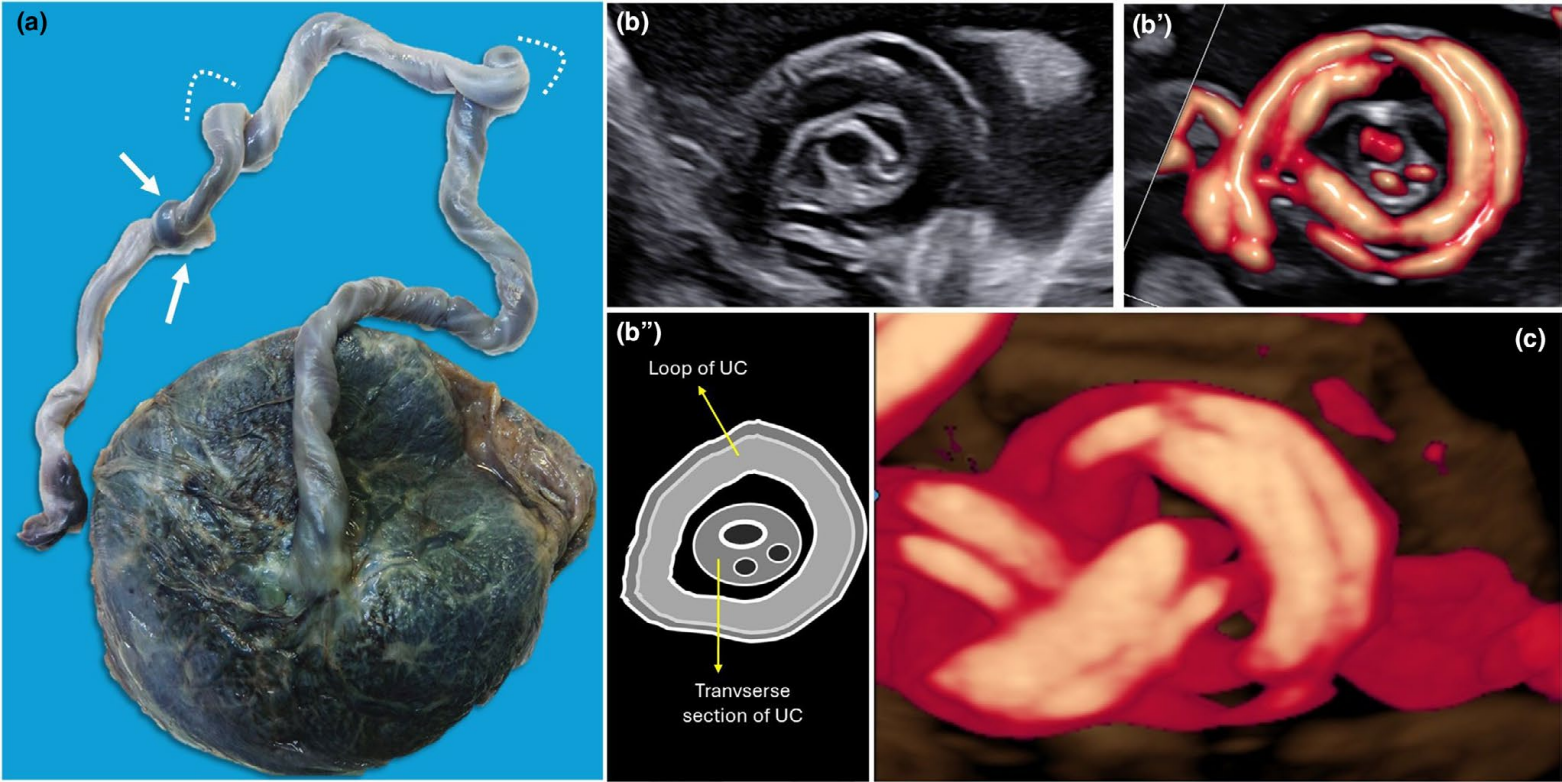
Macroscopic and ultrasound umbilical cord true knot. (a) Macroscopic aspect of the true knot (arrows) in the umbilical cord, also affected by pathological fixed torsions (dotted curved lines). (b) Two‐dimensional ultrasound using gray‐scale (b) color Doppler (b') showing the “hanging noose sign”: A transverse section of the umbilical cord is surrounded by a loop of the umbilical cord, as shown in the drawing (b"). This sign is useful to suspect the presence of a true knot. (c) Three‐dimensional ultrasound appearance of a true knot using power Doppler.

Most intrauterine detections of true knots are from accidental observations during routine scans. During ultrasound, two adjacent cord segments might appear in a fixed crossing, suggesting entanglement: the visualization of one cord segment encircled by another, known as the “hanging noose sign” (Figures 3 and 4), is specific for a true knot.^20^

**FIGURE 5:**
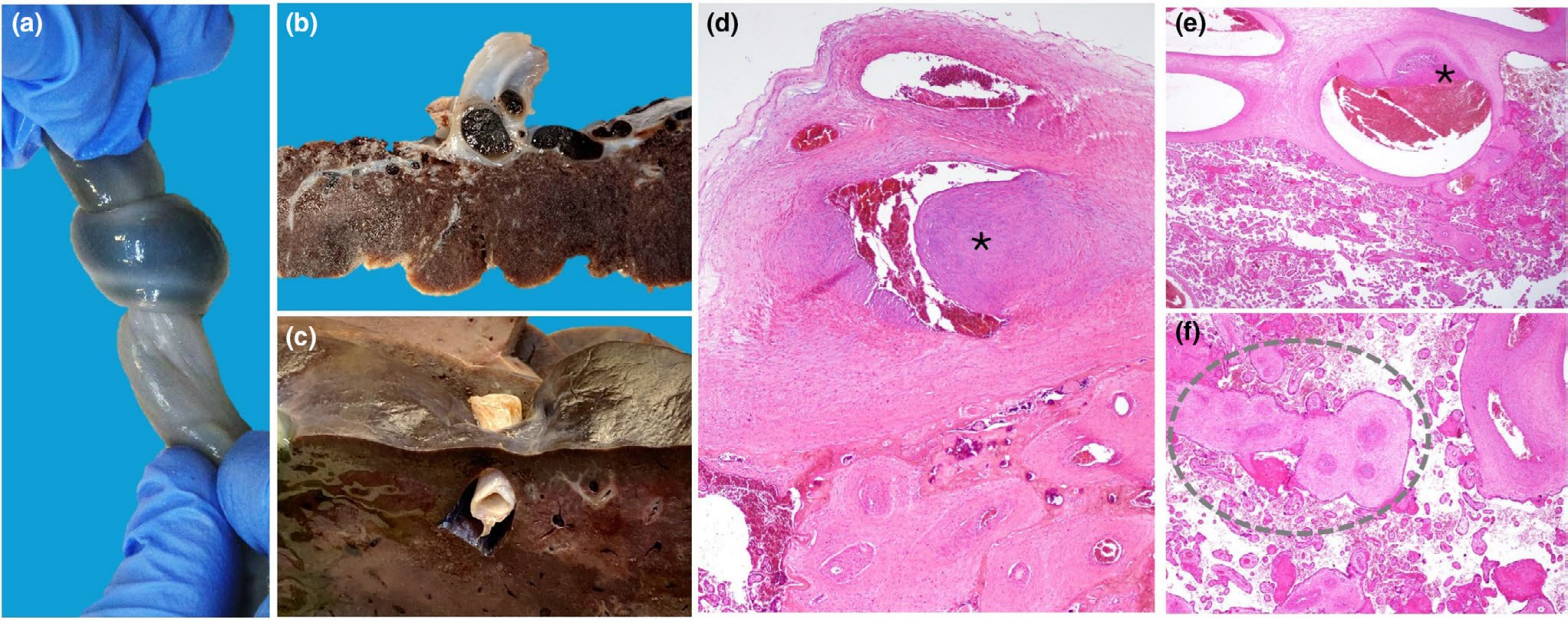
Placental and fetal effects of umbilical cord true knot. (a) umbilical cord true knot. Note the difference in the color of the umbilical cord before and after the knot, as a sign of blood flow interruption. (b) Full‐thickness section of placental parenchyma, collected close to the umbilical cord insertion site. Note the vascular ectasia of the vessel on the cut surface. (c) Section of fetal liver showing the empty umbilical vein due to fetal blood flow obstruction. (d) Section of the upper third of the placenta showing an intramural cushion of fibrin deposition on a vein (overlaid by two arteries) of the chorionic vessels (black asterisk) (H&E; ×4). (e) Nodular myxofibromatous endothelial cushion with fresh on top layer of fibrin deposition (black asterisk) (H&E; ×4). (f) Stem vessels obliteration – also called “fibromuscular sclerosis” (circle) (H&E; ×10). These histological findings might provide supportive evidence of a possible association between cord pathology and the mechanisms contributing to fetal death. H&E, hematoxylin and eosin.

After delivery, at gross evaluation, signs of umbilical cord congestion can be noted, as well as differences in the diameter or color (Figures 3 and 5) of the portion of the cord proximal and distal to the true knot. The histological evaluation can reveal mural thrombosis of the umbilical vein at the knotting site (Figure 3) and venous distension in the portion of the vessel preceding the true knot.^21^ In the placenta, venous stasis can lead to marked distension and thrombosis of the chorionic plate vessels (Figure 5).^21^ Endothelial cushions can be observed (Figure 5) with villous stromal karyorrhexis, suggesting an obstruction that occurred over a prolonged period before death.^15^ Villous edema (Figure 3) can also be observed in these stillborn cases, representing a non‐specific sign of obstruction and alteration of blood pressure.^16^ It is thought that increased venous pressure leads to transudation of fluid into the interstitial space of the terminal villi, leading to the development of villous edema.^16^

#### 3.2.4 Possible strategies for prevention or surveillance

Regarding the possibility of stillbirth prevention, there are no evidence‐based guidelines. The clinical management of pregnancies with the incidental finding of a true knot is challenging and generates a dilemma for obstetricians.^22^ Routine antenatal surveillance is not effective in predicting sudden knot tightening and fetal demise. However, alterations in the umbilical artery Doppler waveform, particularly notching caused by ≥75% luminal occlusion, have been proposed as markers of progressive constriction and an increased risk of stillbirth, warranting consideration of delivery.^23^ Such monitoring might detect gradual tightening before death, thereby enabling preventive intervention. Although vaginal delivery remains feasible, cesarean section rates are approaching 100%, largely driven by parental anxiety, clinician concern, and medico‐legal considerations.^24^

### 3.3 Abnormal cord insertion: Velamentous cord insertion

#### 3.3.1 Definition and prevalence

**FIGURE 6:**
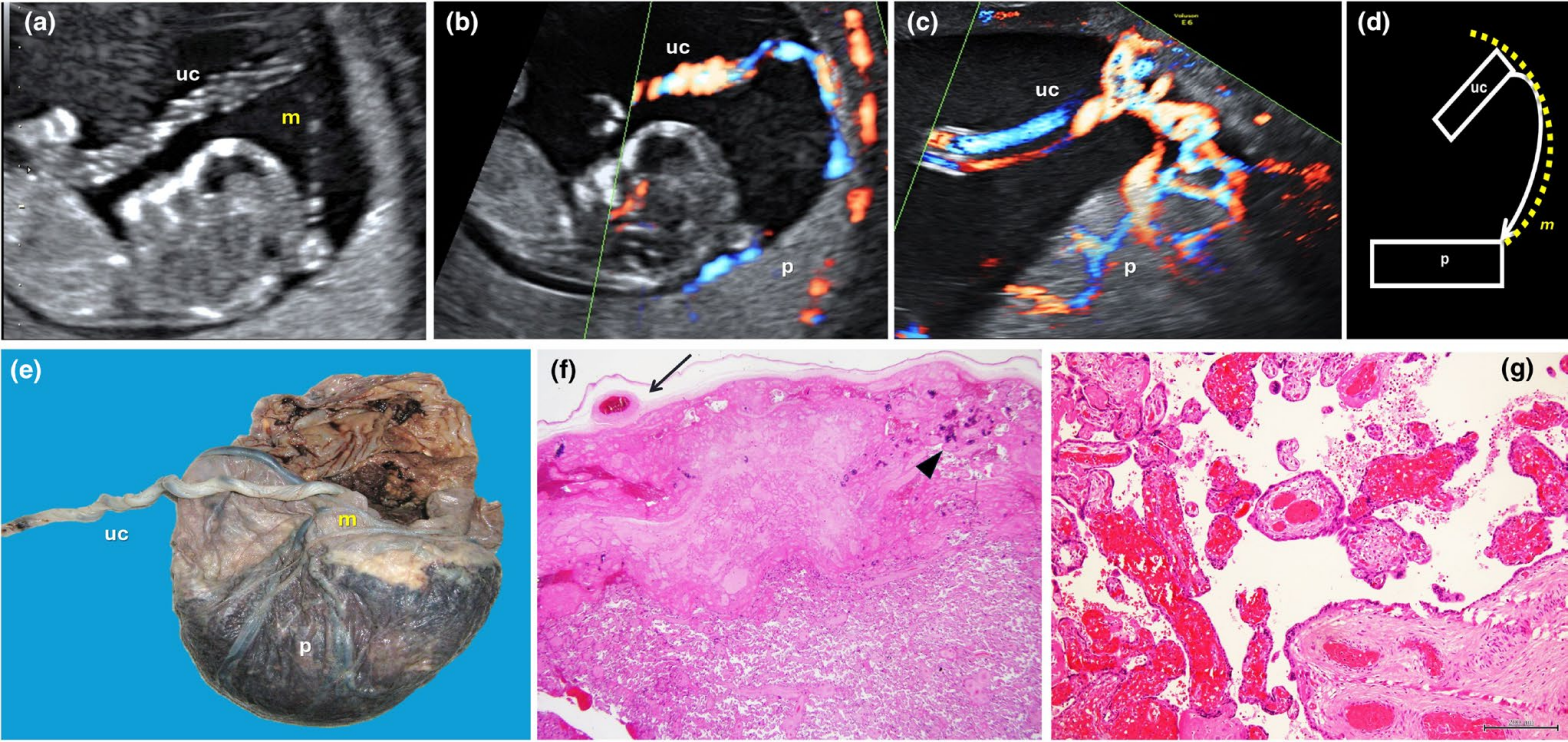
Velamentous cord insertion: ultrasound appearance, macroscopic aspects, and histological signs of the effects of umbilical blood flow obstruction. (a–c) Ultrasound features: Gray‐scale (a) and color Doppler (b, c) ultrasound scans of velamentous cord insertion (a, b: First trimester; c: Second trimester). (d) Drawing summarizing the ultrasound aspect of the velamentous cord insertion: Fixed segment of the umbilical cord (uc) from which the vessels diverge from each other (curved arrow), inserting into the membranes (m; curved dashed line) before entering the placental tissue (p). (e) Macroscopic aspect of velamentous cord insertion. m, membranes; p, placenta; Uc, umbilical cord. (f) Effects of umbilical blood flow obstruction: An avascular area of the placenta, located under the obstruction of blood flow in the chorion vessel (arrow). Note the widespread presence of dystrophic calcification (particularly on the right, arrowhead) as a possible suggestion of subacute/chronic fetal hypoxia (H&E; ×4). (g) Effects of umbilical blood flow obstruction: Intravillous hemorrhage, reflecting an acute obstruction of peripheral fetal venous blood flow (H&E; scalebar 200 μm). The coexistence of lesions shown in (f) and (g) suggests that the obstruction of the blood flow develops during more than one episode before the fatal event. H&E, hematoxylin and eosin.

Velamentous cord insertion, defined as umbilical cord attachment outside the placental disc with intramembranous vessels lacking Wharton's jelly (Figure 6), occurs in approximately 1% of pregnancies (0.5%–1.69% in singletons; 10‐fold higher in twins).^25^, ^26^ This anomaly is an established risk factor for adverse outcomes.^12^ A recent meta‐analysis demonstrated a fourfold increased stillbirth risk (RR 4.12; 95% CI 1.92–8.87),^27^ while other studies found an adjusted risk exceeding ninefold (adjusted OR [aOR] 9.56; 95% CI 6.76–13.5).^28^

#### 3.3.2 Mechanism of death

Fetal death in velamentous insertion can primarily result from compression and thrombosis of intramembranous vessels, which, lacking support from placental tissue and Wharton's jelly, are highly vulnerable to obstruction. In this context, the fetus is both victim and villain: its own movements might exacerbate compression, leading to stasis, interrupted flow, thrombosis, and ultimately death.^12^ Vessel length, defined as the distance from cord insertion to the placental edge, correlates with vulnerability^12^: the longer the distance, the higher the risk.

Another mechanism that could contribute to stillbirth is placental hypoperfusion related to the mechanical damage of the umbilical cord vessels.^27^ This hypoperfusion can also cause fetal growth restriction; indeed, velamentous cord insertion is a potential risk factor for fetal undergrowth.^24^, ^29^ Notably, severe undiagnosed fetal growth restriction can yield intrauterine fetal death.^27^

#### 3.3.3 Relevant diagnostic findings

Placental cord insertion can be visualized early in pregnancy, with velamentous insertion appearing outside the disc in a fixed location (Figure 6).^26^ Intramembranous vessel branching is detectable with color Doppler (Figure 6), showing vessels running parallel to the uterine wall from insertion to placental margin.^26^ Second‐trimester ultrasound demonstrates high accuracy for detecting velamentous insertion (94.4%–100%), with color Doppler achieving sensitivity 100%, specificity 99.9%, and positive predictive value 85.7%.^30^, ^31^

The histological placental signs of fetal blood flow obstruction are the same as previously mentioned (Figure 6).

#### 3.3.4 Possible strategies for prevention or surveillance

Although velamentous cord insertion can be readily detected by ultrasound, its management remains largely unstandardized worldwide.^30^ Awareness of the condition should prompt monitoring, including fetal growth assessment, to identify and manage growth restriction and potentially improve outcomes. The European association of perinatal medicine suggest individualized follow‐up during pregnancy and tailored obstetric management in cases affected by velamentous cord insertion.^32^ Irrespective of the fetal growth, the American College of Obstetricians and Gynecologists advocated weekly antenatal fetal surveillance starting at 36 weeks of gestation in cases affected by velamentous cord insertion in the outpatient setting with the aim of improving fetal outcome and reducing the risk of stillbirth.^33^

### 3.4 Abnormal cord insertion: Furcate cord insertion

#### 3.4.1 Definition and prevalence

**FIGURE 7:**
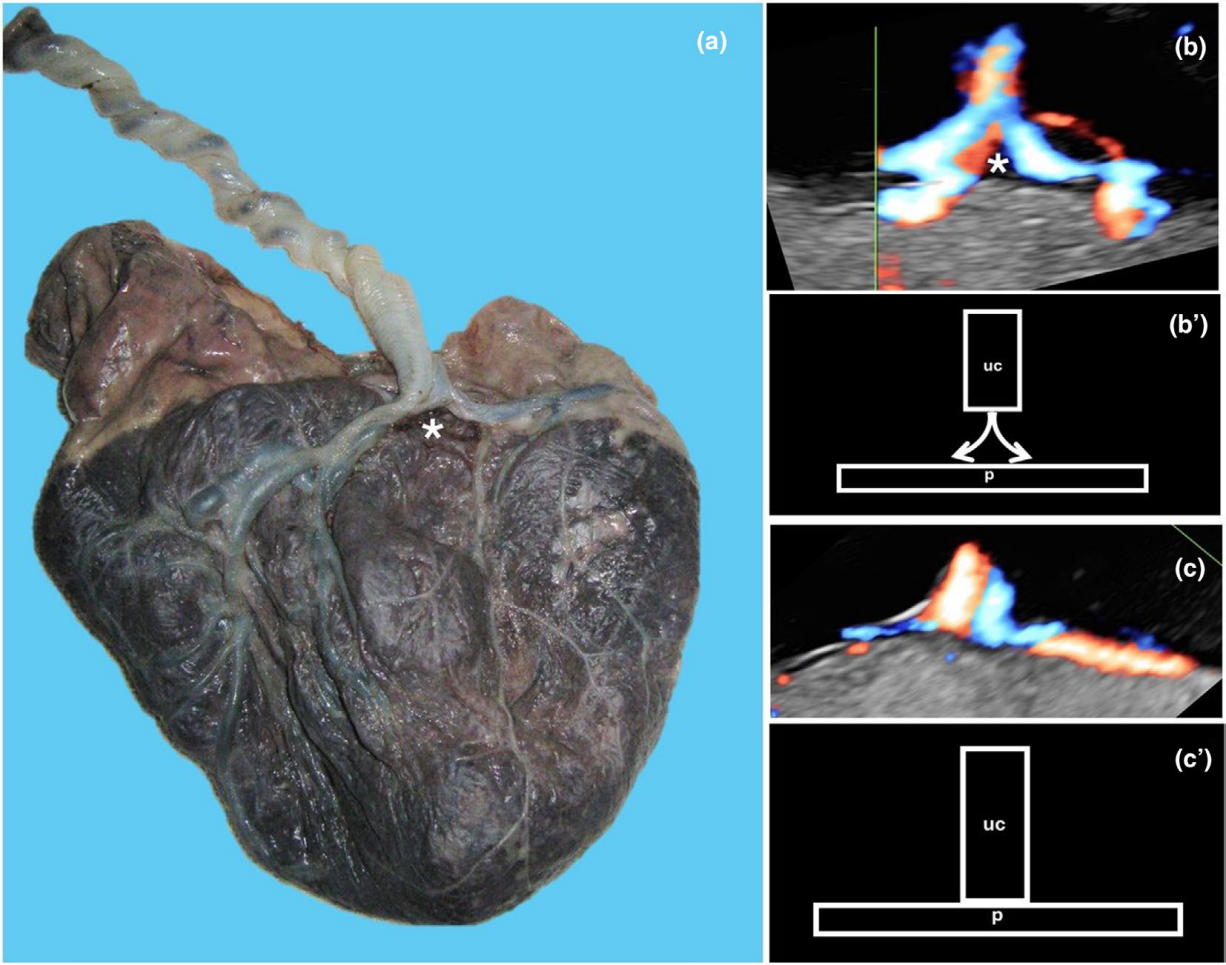
Furcate umbilical cord insertion. (a) Macroscopic appearance of furcate insertion of the umbilical cord in which the cord divides into two branches before inserting onto the placental surface (asterisk). Note also that, in this case, the umbilical cord inserts peripherally into the placental disc. (b) Color Doppler ultrasound scan (b) and drawing (b') of the dicotomic direction of the umbilical vessels. (c) Color Doppler ultrasound scan (c) and drawing (c') of normal umbilical cord insertion.

A peculiar type of abnormal cord insertion into the placenta is the furcate cord insertion (Figure 7), a rare condition that affects 0.16% of pregnancies.^34^ In fact, its incidence is overlooked in most cases, mainly due to the lack of knowledge of the meaning of this abnormality.^34^ In this condition, the three umbilical vessels separate from each other before reaching the placental disc, indeed increasing their vulnerability to compression and obstruction.^12^

Most cases with furcate cord insertion are uneventful,^34^ but exposed vessels are more prone to mechanical injury, increasing stillbirth risk.

#### 3.4.2 Mechanism of death

Fetal death might result from thrombosis due to compression of the furcated vessels,^12^ or, rarely, from rupture of an ectatic segment within these vessels.^12^, ^35^

#### 3.4.3 Relevant diagnostic findings

Furcate cord insertion can be detected by the ultrasound scan, observing the dichotomic direction of the umbilical blood flow in the vessels near their insertion into the placental disc^18^ (Figure 7).

#### 3.4.4 Possible strategies for prevention or surveillance

No formal suggestions about the prevention of antepartum fetal death are available. Recent data suggest that the prenatal ultrasound detection of furcate cord insertion implies personalized counseling on risks and pregnancy management.^34^ Some authors considered early‐term delivery to reduce the risk of sudden fetal death.^36^ However, the paucity of evidence on this lesion warrants careful weighing of the potential harms of overtreatment against the risks of expectant management.

### 3.5 Segmental dilatation of the umbilical vein

#### 3.5.1 Definition and prevalence

Pathological zonal dilatation of a vessel with a weakened wall is referred to as an aneurysm. Usually, an aneurysm affects the vein. It is a relatively rare lesion. Overall, aneurysms were reported in less than 2% of placentas, with a twice increased prevalence in placentas from stillbirth.^37^ Other investigations reported a rarer prevalence of aneurysmatic dilatation, occurring in one case per 2300 births.^38^

#### 3.5.2 Mechanism of death

**FIGURE 8:**
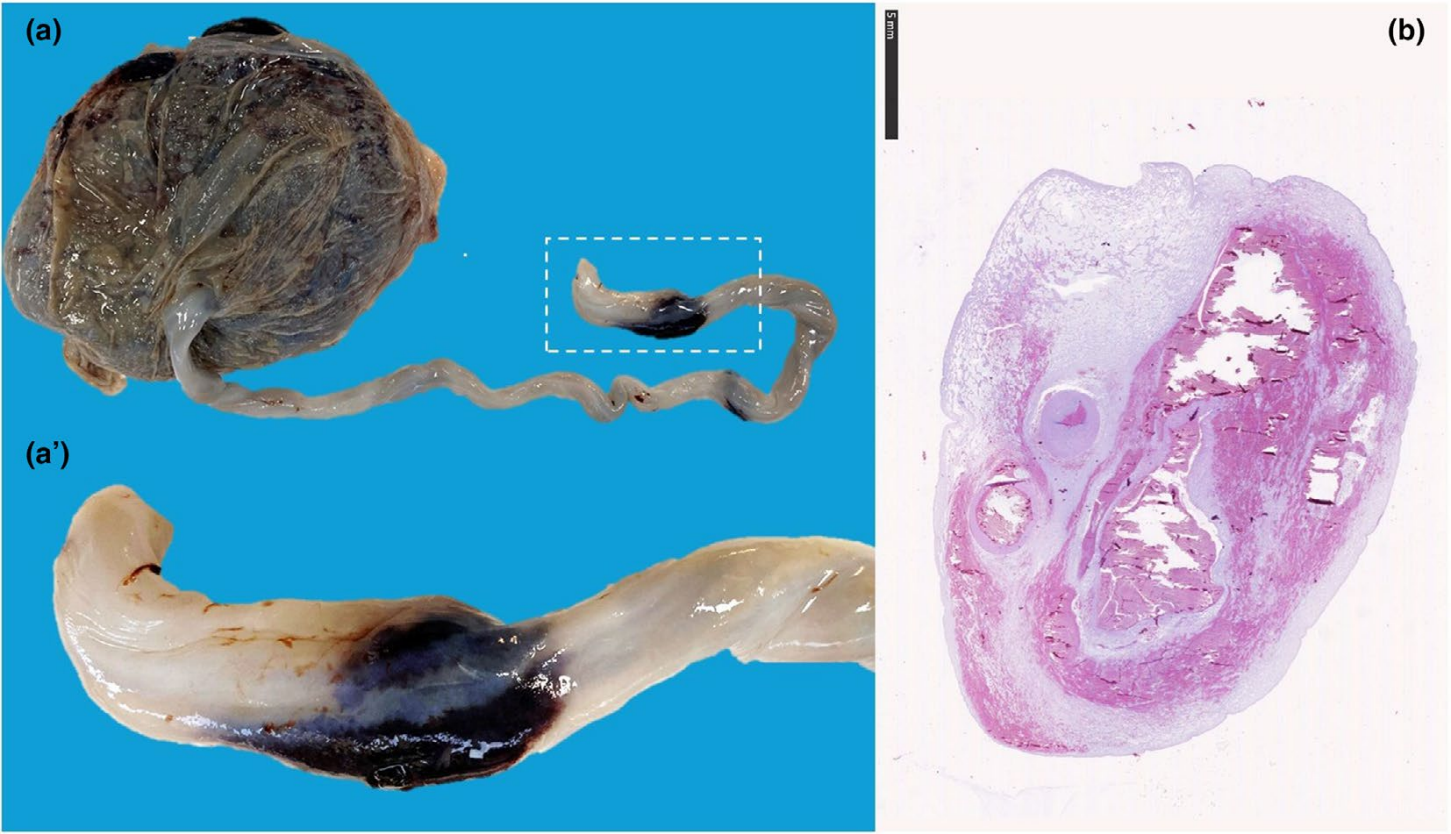
Effects of aneurysm. (a–a') Macroscopic appearance of umbilical cord hemorrhage related to rupture of the vascular wall in case of aneurysmal umbilical cord venous malformation. (b) Transverse section of the umbilical cord affected by aneurysmal dilatation of the umbilical vein, showing a small thin rim of the vessel wall. Note the blood within the distended vein as well as within Wharton jelly (H&E; scale bar 5 mm). These macroscopic and histological findings offer supportive evidence implicating rupture of the aneurysm as a potential mechanism leading to fetal demise. H&E, hematoxylin and eosin.

The mechanism leading to fetal death can be related to fetal cardiac insufficiency due to volume overload, causing fetal heart failure^38^; in other cases, stillbirth is related to the rupture of the aneurysm (Figure 8) due to the weakness of the dilated wall; vascular thrombosis into the dilated segment of the vessel can be another mechanism that causes fetal death.^39^

#### 3.5.3 Relevant diagnostic findings

**FIGURE 9:**
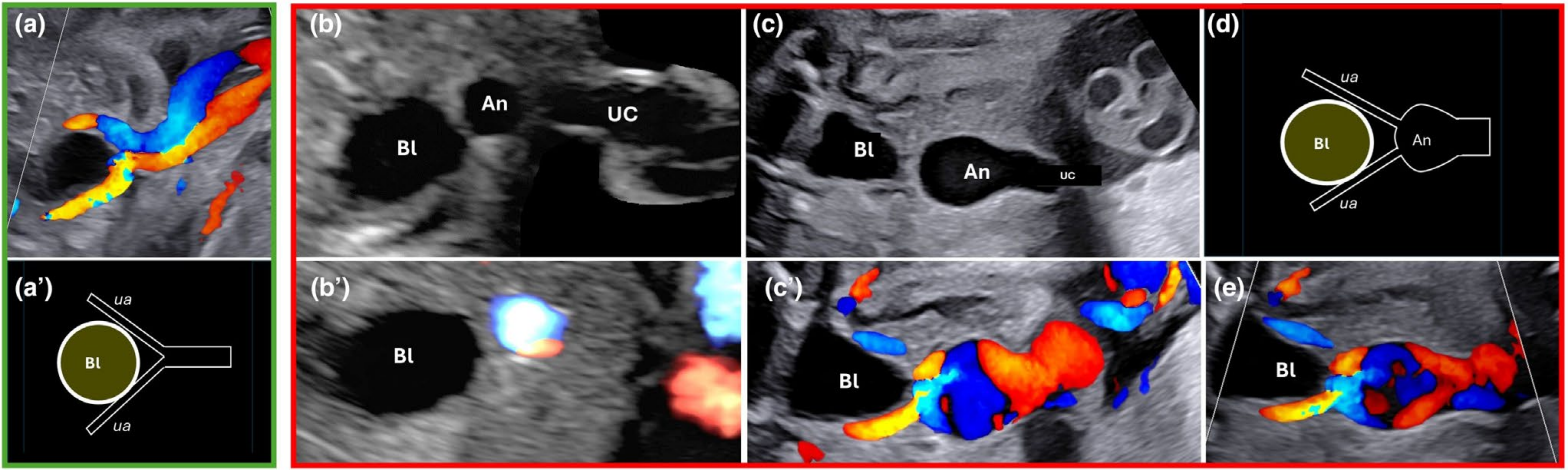
Intrabdominal aneurysm of the umbilical vein. (a) Color Doppler ultrasound and (a') schematic representation of the normal appearance of the intra‐abdominal segment of the umbilical cord. (b, c) aneurysmal dilatation of the umbilical vein. Gray‐scale (b, c) and color Doppler (b', c') ultrasound scans of two different cases affected by an aneurysm of the umbilical vein. Note the round‐shaped anechoic cystic‐like structure in b and the oblong cystic‐like structure in c. (d) Schematic representation of aneurysmal dilatation of the umbilical vein. (e) Another example of aneurysmal dilatation of the umbilical vein. Note the bidirectional turbulence flow in the dilated segment. An, aneurysm; Bl, bladder; Ua, umbilical artery; Uc, umbilical cord.

Anatomically, the vessel shows an abnormal focal dilatation. This aneurysmal enlargement can cause an alteration of the fetal blood flow.^40^ Frequently, but not always, the dilatation occurs in the intrabdominal portion of the umbilical vein (Figure 9). Since the umbilical blood flow increases through gestation, the risk of adverse hemodynamic effects of the aneurysms increases with the gestational age.^37^

There are no universal criteria of definition for the antenatal ultrasound diagnosis of the aneurysm of the umbilical vein. Ultrasound transverse gray‐scale images can show an anechoic oblong, oval‐shaped, or rounded cystic‐like structure in the fetal abdomen near the abdominal wall (Figure 9); in other cases, the dilatation can be seen between the abdominal wall and the liver.^41^ A previous report suggests that 9 mm is the threshold above which the vein diameter is considered abnormally large.^41^ Another criterion used for the diagnosis of the aneurysm is the ratio between the transverse diameter of the extrahepatic umbilical vein and the transverse diameter of the intrahepatic component; when the extrahepatic diameter is greater than 1.5 times the intrahepatic, the former is aneurysmatic. Other authors considered dilated the vessel when it measures more than two standard deviations of the mean for gestational age^42^; however, referral data are scanty. The use of color Doppler improves the diagnosis, allowing for the ruling out of other non‐vascular fluid cystic mass detectable in this area.^43^ Color Doppler examination shows vascular flow into the cystic structure^41^ (Figure 9). In the presence of a large dilatation (above 12 mm^25^) and bidirectional turbulence flow, the risk of thrombosis^23^ and, therefore, the risk of fetal death^25^ increase.

#### 3.5.4 Possible strategies for prevention or surveillance

No evidence‐based guidelines exist for preventing stillbirth in umbilical cord aneurysms. Reports recommend close monitoring from diagnosis to delivery to detect fetal distress.^38^, ^40^, ^43^, ^44^, ^45^ Ultrasound can assess size, clot formation, and signs of fetal decompensation (pericardial effusion, edema, hydrops).^38^ However, thrombus formation is unpredictable and can cause sudden fetal death, even with close surveillance.^38^, ^45^ Therefore, parents should be counseled that monitoring could reduce but does not eliminate the risk.^45^

Current evidence advises against preterm delivery when fetal surveillance is normal, recommending prompt delivery only if ultrasound shows a clot or signs of fetal decompensation.^38^ Early‐term delivery could be considered^40^, ^44^, ^45^ where facilities for emergency cesarean section are available, upon the occurrence of concerning events during labor.^44^ Shared decision‐making should be undertaken with parents, balancing the risk of early‐term induction of labor and the risk of sudden, unpredictable fetal death in prolonged pregnancy.

### 3.6 Umbilical cord stricture

#### 3.6.1 Definition and prevalence

Umbilical cord stricture is a rare condition of focal deficiency of Wharton's jelly,^12^ with localized increased collagen at the site and a decreased umbilical cord diameter in relation to the diameter of the remaining length of the umbilical cord.^46^

#### 3.6.2 Mechanism of death

Localized scarcity of Wharton's jelly creates a weak segment of the cord, which might act as a pivot around which the fetus can rotate.^23^ The rotation around the stricture increases the risk of vascular restriction, reducing the fetal blood flow up to blood flow interruption and increasing the likelihood of fetal death.^46^

#### 3.6.3 Relevant diagnostic findings

**FIGURE 10:**
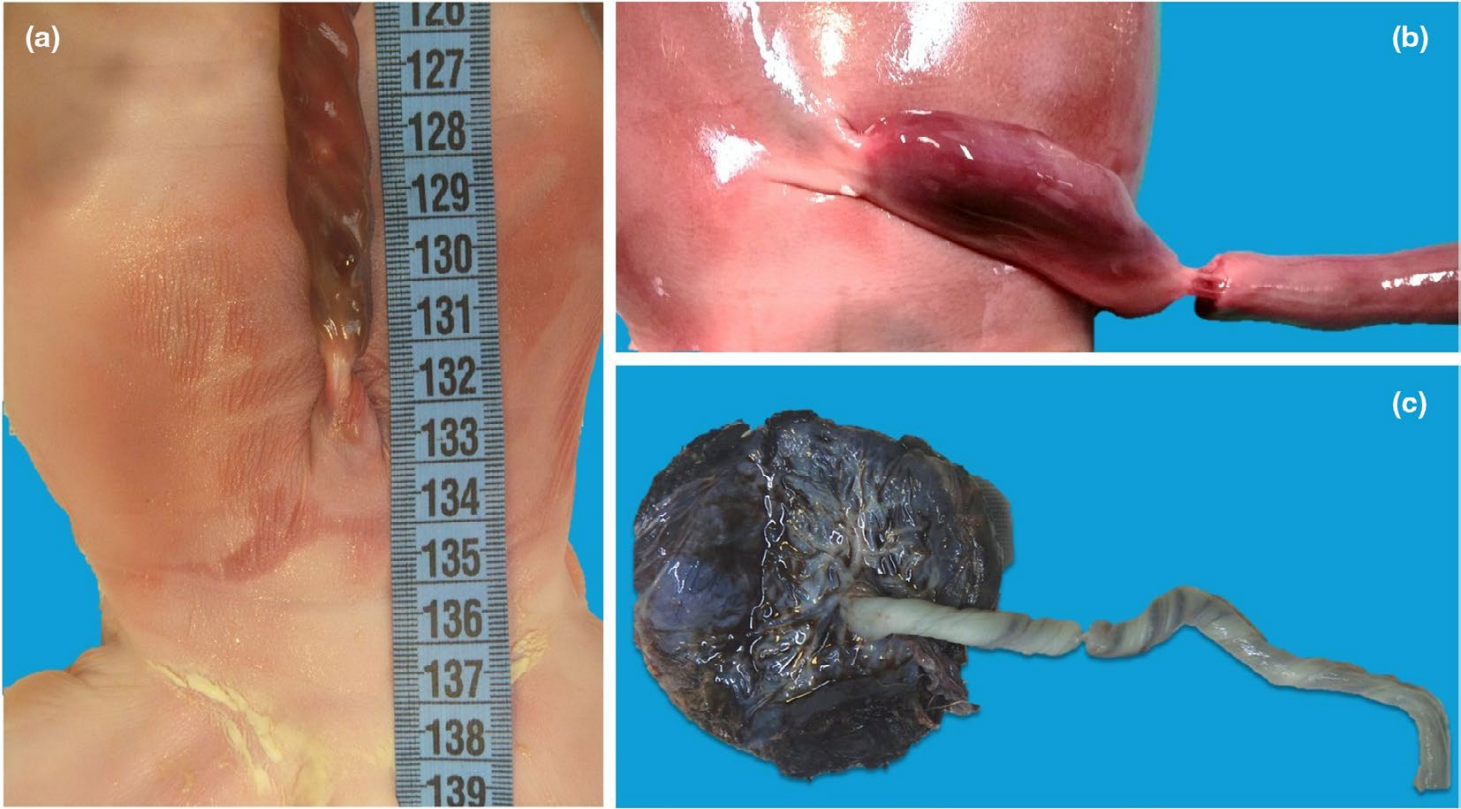
Umbilical cord stricture. Umbilical cord stricture affecting the fetal site (a) of cord insertion or free loop segment of the cord, near the fetal site of insertion (b) or near the placental site of insertion (c). The stricture might lead to blood flow reduction up to interruption, increasing the risk of stillbirth, as occurred in the presented cases.

Usually, the umbilical cord stricture is seen at the fetal end of the cord (Figure 10).^12^ However, the stricture can occasionally occur in other segments of the cord. When located far from the fetal end, it is more challenging to detect during pregnancy and is usually observed just after delivery. The stricture can occasionally affect a segment of the umbilical cord far from the fetal end (Figure 10). Notably, it is important to distinguish true umbilical cord stricture from common postmortem maceration artifacts at the fetal end^12^ and to interpret any stricture within the overall context of the case.^21^

Histologically, vascular thrombosis can be detected in the coarcted vessels. The demonstration of thrombosis in the vessel is hampered in cases with fetal death to distinguish between the postmortem artifact from a real stricture that causes stillbirth.^21^

#### 3.6.4 Possible strategies for prevention or surveillance

Although it is an uncommon condition, it is important to keep it in mind because a risk of recurrence has been observed in subsequent pregnancies.^47^

Table 1 summarizes the abovementioned umbilical cord conditions, the relationship to fetal death, and potential strategies of stillbirth prevention.

**TABLE 1:** Umbilical cord conditions and strategies for stillbirth prevention.

Umbilical cord abnormalities	Definition	Risk of stillbirth	Ultrasound signs	Potential mechanisms of death	Potential strategies of prevention	Quality of the suggestion
Multiple nuchal cord	Multiple complete loops of the umbilical cord around the whole circumference of fetal neck	Multiple nuchal cord: OR of 2.36; 95% CI 0.99–5.62, in comparison with cases without nuchal cord Four loops of nuchal cord: OR 13.40; 95% CI 1.12–160.34	Divot sign: Sagittal section of fetal neck with scalloping of the fetal subcutaneous tissue that appears as indented due to the nuchal cord compression on the fetal subcutaneous tissue below the nuchal cord	Blood flow interruption due to obstructive mechanism of compression and/or traction of the umbilical vessel and/or Obstruction of the jugular venous return leading to congestion of cerebral and meningeal vessels, and causing intracranial hemorrhage and/or Compression of the carotid arteries, raising blood pressure, leading to cardiac remodeling	No need for intervention in case of single nuchal cord (benign condition) Increased antepartum fetal surveillance in case of multiple nuchal cord Consider early‐term delivery in complicated cases	Low (*Commentary*)^8^ (*Review of the literature*)^19^
True knot	Entwining of a segment of the umbilical cord around itself due to fetal get in through a loop of the cord	OR of 3.96; 95% CI 1.85–8.47 (up to aOR 15.46, 95% CI 9.30–25.70) in comparison with cases without true knot	Hanging noose sign: transverse section of the umbilical cord surrounded by a loop of umbilical cord	Blood flow interruption due to tightening of the knot	Antenatal surveillance is not effective in predicting sudden knot tightening Close surveillance of markers of progressive constriction in the umbilical artery Doppler waveform	Low (*Retrospective observational study and review of the literature*)^24^
Velamentous cord insertion	Umbilical cord attachment outside the placental disc with intramembranous vessels lacking Wharton's jelly	aOR 9.56; 95% CI 6.76–13.5 in comparison with cases without velamentous cord insertion	Umbilical cord insertion outside the placental disc in a fixed location. Color Doppler findings: vessels running parallel to the uterine wall from insertion to placental margin	Blood flow reduction and interruption due to compression and thrombosis of intramembranous vessels Placental hypoperfusion (related to the mechanical compression of the umbilical cord vessels) could determine fetal growth restriction	Fetal growth assessment, to identify and manage fetal growth restriction Individualized follow‐up during pregnancy and tailored obstetric management (suggested by EAPM) up to Weekly antenatal fetal surveillance starting at 36 weeks of gestation (suggested by ACOG)	*Moderate* (*Position statement EAPM*)^32^ (*Committee opinion ACOG*)^33^ (*Metanalysis*)^27^
Furcate cord insertion	Insertion of the cord with the umbilical vessels that separate each‐other before reaching the placental disc	Undetermined (rare condition <1% of pregnancies)	Dichotomic direction of the umbilical blood flow in the vessels near their insertion into the placental disc	Thrombosis of the furcated vessels due to vessels compression	No formal suggestions Early‐term delivery considered by some authors to reduce the risk of sudden fetal death	Low (*Opinion from Retrospective cohort study case series*)^34^ (*Opinion from case report*)^36^
Aneurysm	Zonal dilatation of a vessel with a weakened wall	Undetermined (rare condition, <2% of placentas, twice in stillbirth)	Anechoic oblong, oval‐shaped, or rounded cystic‐like structure in the fetal abdomen near the abdominal wall or between the abdominal wall and the liver. Color Doppler findings: turbulent vascular flow into the cystic‐like structure	Fetal cardiac insufficiency due to volume overload, causing fetal heart failure. or Rupture of the aneurysm. or Vascular thrombosis into the dilated segment of the vessel.	Close monitoring from diagnosis to delivery, aimed at detecting fetal distress and signs of fetal decompensation (pericardial effusion, edema, hydrops). Thrombus formation is unpredictable. It can cause sudden fetal death, even with close surveillance Early term delivery could be considered	Low (*Case report*)^38^ (*Case series*)^40^ (*Case report*)^45^ (*Case series*)^42^
Umbilical cord stricture	Focal deficiency of Wharton's jelly with localized increased collagen in such site and a decreased umbilical cord diameter in relation to the diameter of the remaining length of umbilical cord	Undetermined (rare condition)	Segment of the umbilical cord markedly thinner than the surrounding. Low cross‐sectional area of the umbilical cord due to reduction of Wharton's jelly	Blood flow reduction and interruption due to vascular restriction	No formal suggestions. Attention in subsequent pregnancies: recurrence has been observed	Very Low (*Case report*)^47^

Abbreviations: ACOG, American College of Obstetricians and Gynecologists; aOR, adjusted odds ratio; CI, confidence interval; EAPM, European association of perinatal medicine; OR, odds ratio.

## 4 OTHER COMMON UMBILICAL CORD CONDITIONS LACKING ACTIONABLE PREVENTIVE MEASURES

### 4.1 Definition and prevalence

**FIGURE 11:**
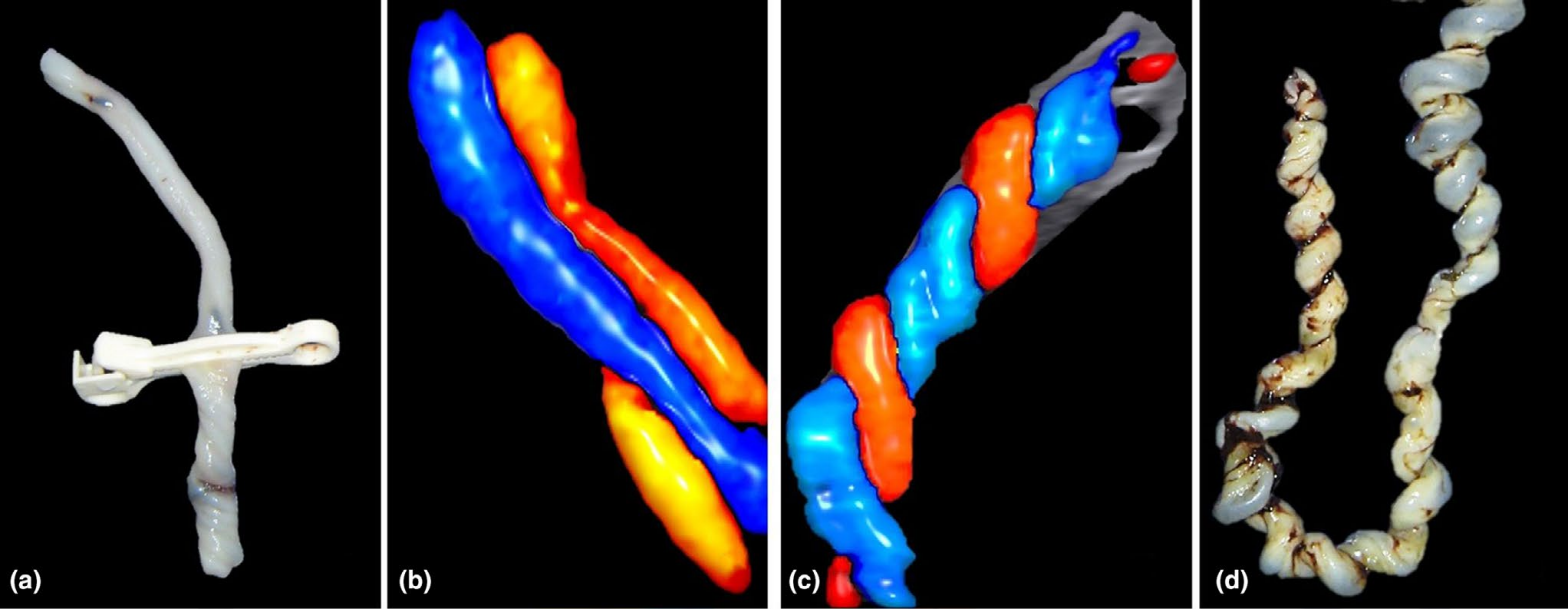
Abnormal coiling. (a, b) Macroscopic and ultrasound appearance of hypocoiled or (c, d) hypercoiled umbilical cord. Although these umbilical cords' abnormal cooling might affect cord blood flow and fetal hemodynamics, the heterogeneity of existing studies prevents the establishment of definitive causal links or evidence‐based preventive strategies.

**FIGURE 12:**
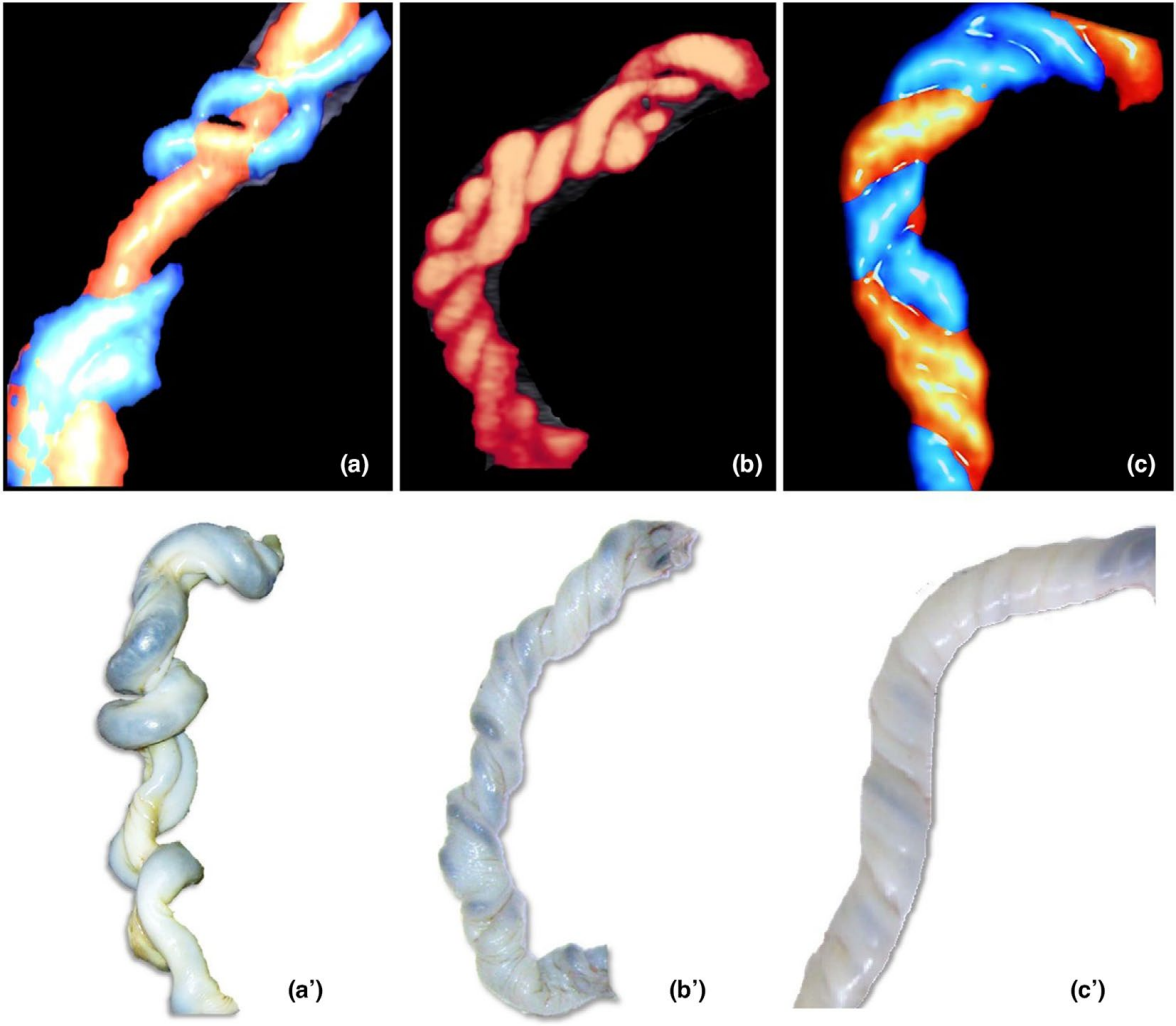
Ultrasound and macroscopic umbilical cord features. (a) Umbilical cord with low coiling. (a') Vascular dilatation as a result of obstruction in the umbilical cord with low coiling. (b) Ultrasound appearance of a hypercoiled umbilical cord showing an undulating appearance. (b') Macroscopic appearance of hypercoiled umbilical cord showing an undulating appearance. (c) Ultrasound appearance of hypercoiled umbilical cord showing rope appearance. (c') Macroscopic appearance of hypercoiled umbilical cord showing rope appearance. While these structural anomalies of the umbilical cord can influence fetoplacental circulation and affect fetal hemodynamics, current evidence remains insufficient to inform targeted preventive measures.

Other umbilical cord characteristics, such as abnormal length and abnormal coiling (Figures 11 and 12), were potentially associated with adverse fetal outcomes.^11^, ^12^, ^21^, ^48^, ^49^

A hypercoiled umbilical cord shows excessively twisted vessels, whereas a hypocolided cord appears as an extremely smooth or untwisted cord.

Abnormalities in umbilical cord length and in the coiling of the vessels can affect fetal circulation. However, studies on the potential association between stillbirth, abnormal length, and abnormal coiling of the umbilical cord used different thresholds and different populations at enrollment. Therefore, recent studies cannot provide specific prevalence or conduct meta‐analyses to assess the association with these abnormal umbilical cord features.^5^, ^50^

### 4.2 Mechanism of death

Regarding the mechanism of fetal damage, it has been suggested that excessive umbilical cord length can impair the fetal circulation by increasing the resistance to blood flow.^11^ According to Poiseuille's law, the vessel resistance is directly proportional to the length of the vessel and the viscosity of the blood. In this view, comparing two vessels with equal diameter but one twice the length of the other, the flow resistance is twice that of the former.

Abnormal coiling can cause an intrinsic deformation of the cord, increasing the risk of stricture, torsion, and compression, increasing in turn the risk of blood flow obstruction.^11^

### 4.3 Relevant diagnostic findings

**FIGURE 13:**
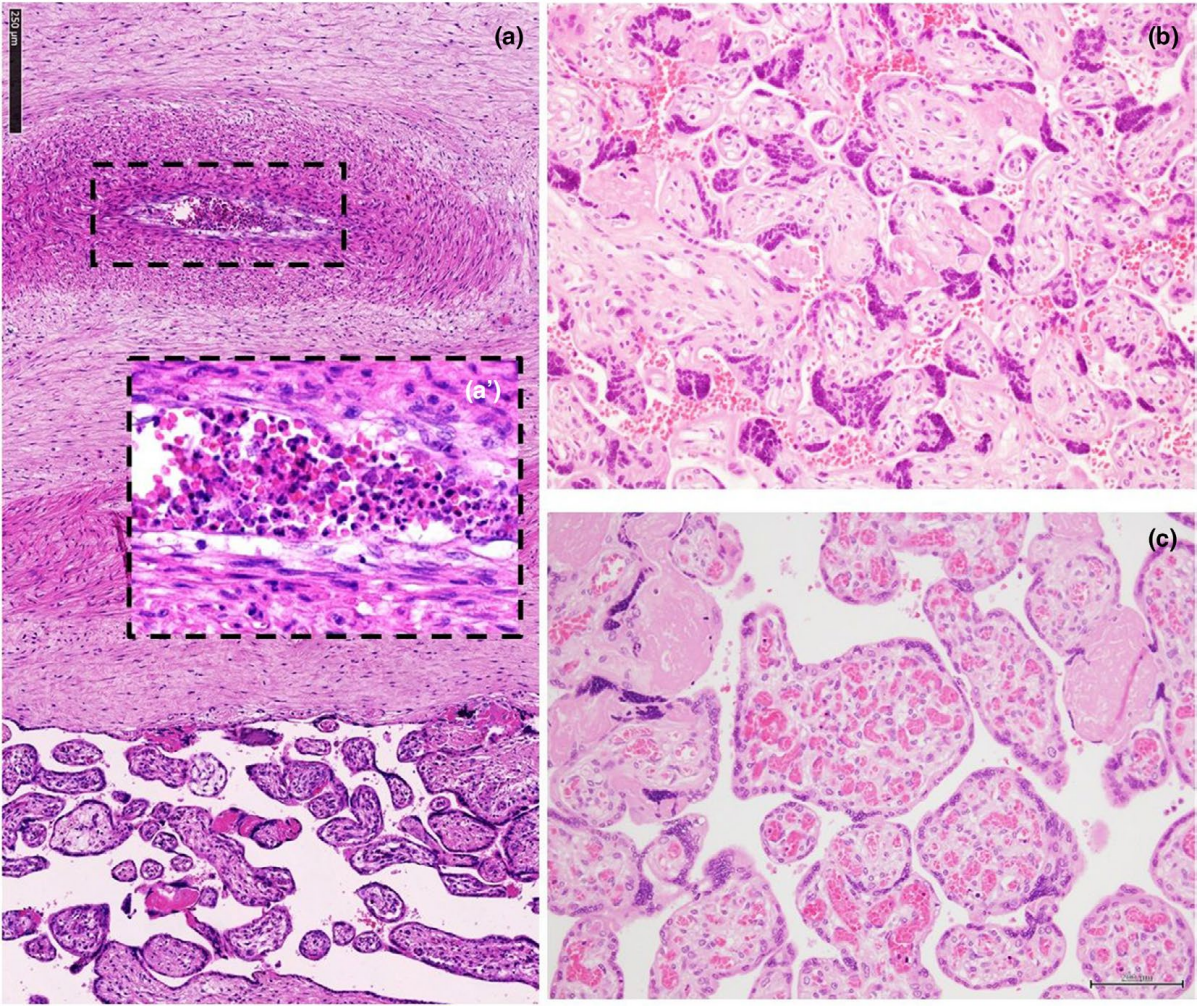
Placental histological non‐specific signs of hypoxia. (a–a') nucleated red blood cells in the fetal circulation, observed in the placental villous vessels (H&E; scalebar 250 μm). (b) Increased number of syncytial knots. (c) Chorangiosis (H&E; scalebar 200 μm). These features are not specific to umbilical cord abnormalities but might also occur in various conditions associated with intrauterine hypoxia; their presence indicates a vital process with chronic temporal evolution. H&E, hematoxylin and eosin.

Although there have been no direct experiments on the human umbilical cord, the increased resistance in the umbilical cord could increase the perfusion pressure to cause fetal heart remodeling: cardiac enlargement and hypertrophy can be seen in infants with long cords.^21^ Histological placental signs of fetal hypoxia could be observed in cases with an excessively long umbilical cord; that is, nucleated red blood cells, chorangiosis, and increased syncytial knots (Figure 13). Moreover, histological placental signs of altered blood flow could be observed; that is, vascular thrombi, vascular cushions‐intramural fibrin deposition, and/or signs of obstruction of venous return from the placenta (e.g., villous capillary congestion).^21^, ^48^

Notably, during the first few hours after delivery, the umbilical cord can shrink up to 7 cm in length.^21^ Therefore, accurately measuring the umbilical cord length in the delivery room after birth is fundamental^21^ to avoid a false underestimation of length in cases potentially affected by a cord accident.

### 4.4 Possible strategies for prevention or surveillance

Recent data report high antenatal ultrasound performance in identifying abnormal umbilical coiling.^50^ However, the high heterogeneity of the medical literature on this topic precluded meta‐analysis.^50^ Consequently, any reasoning regarding the potential for stillbirth prevention remains inconclusive. Moreover, a recent single‐center observational study also evaluated the direction (sinistral or dextral) of the umbilical cord coiling, observing that stillbirths were more associated with sinistral coiling compared to dextral.^51^ However, more extensive and robust studies should be performed to ascertain the clinical relevance of the coiling direction patterns.

## 5 DISCUSSION

### 5.1 Critical synthesis of the evidence

Recent advances in prenatal imaging have markedly improved the detection of both intended and incidental umbilical cord abnormalities. However, the clinical significance of these findings remains uncertain. Some anomalies, such as nuchal cords or true knots, are frequently observed in uncomplicated pregnancies and usually have favorable outcomes.^5^, ^52^, ^53^, ^54^ Otherwise, other conditions, both acquired (multiple nuchal cords and knots) and congenital (abnormal insertion), in certain cases could compromise fetal circulation, raising the likelihood of stillbirth. The association between umbilical cord abnormalities and adverse outcomes is, therefore, more correlational than causative in most cases. Evidence corroboration is required before concluding that a cord accident was the cause of death.^55^ In this context, histopathological placental analysis is mandatory to highlight the pathophysiology of events. Histopathology can demonstrate, when present, features consistent with fetal vascular malperfusion, including thrombosis, stem vessel obliteration, segmental avascular villi, villous stromal‐vascular karyorrhexis, intramural fibrin deposition, and vascular ectasia, all lesions classified as fetal vascular malperfusion.^11^, ^56^, ^57^ However, these lesions are not specific for umbilical cord abnormalities and might occur in stillbirths of multifactorial etiology. A comprehensive case investigation is essential to elucidate the cause of death and the pathophysiological mechanisms underlying stillbirth.

### 5.2 Limitations of the available studies

Interpretation of existing literature is limited by several methodological constraints. Most available data derive from retrospective observational cohorts or case series, which are inherently prone to selection bias, inconsistent diagnostic definitions, and autopsy bias, given that severe or fatal cases are more likely to undergo detailed examination. Further, the heterogeneity of definitions limits comparability among studies. The lack of standardized criteria impedes meta‐analytic synthesis and prevents the establishment of evidence‐based thresholds for clinical action. Randomized controlled trials in this context are ethically and practically unfeasible, resulting in a persistent reliance on expert opinion and indirect inference.

### 5.3 Risks of over‐intervention versus potential benefits of surveillance

Balancing the potential benefits of closer surveillance against the risks of unnecessary intervention remains a major clinical challenge. Communication of an umbilical cord abnormality to expectant parents can heighten maternal anxiety, increase the frequency of medical visits, and potentially lead to premature delivery decisions. While maternal awareness, particularly regarding fetal movements, can contribute to earlier recognition of fetal compromise,^58^ heightened anxiety might prompt unnecessary interventions and result in iatrogenic complications, including preterm birth and cesarean delivery without proven survival benefit. Current evidence does not support systematic preterm delivery^8^, ^36^, ^40^, ^44^, ^45^ and favors an individualized, risk‐based approach, as no strong evidence‐based preventive strategy has demonstrated efficacy in reducing stillbirths attributable solely to umbilical cord abnormalities.

### 5.4 Current clinical implications

At present, clinical management should prioritize judicious ultrasound follow‐up, focusing on fetal well‐being parameters (growth, Doppler velocimetry, and signs of cardiac decompensation) rather than the mere presence of a cord anomaly.^13^, ^14^, ^21^, ^23^, ^24^, ^38^, ^58^ It could be useful to share the arrangement of the management of fetal monitoring. While continuous surveillance cannot prevent sudden cord occlusion, it might help identify progressive fetal deterioration in selected high‐risk cases. Shared decision‐making with parents, grounded in transparent discussion of uncertainties, remains essential. Preventive measures such as early‐term delivery might be considered in specific, individualized scenarios where the risk of late stillbirth outweighs the potential harm of intervention.^3^

### 5.5 Global perspective

The potential preventability of stillbirths associated with umbilical cord abnormalities, as outlined in this review, is intrinsically linked to the availability of advanced ultrasonographic surveillance, an approach largely confined to high‐income settings and currently limited by a low predictive value of prenatal imaging in anticipating the progression of detected abnormalities. In contrast, the global burden of stillbirth is disproportionately concentrated in low‐ and middle‐income countries, where access to such diagnostic modalities remains limited.^59^, ^60^ Within these contexts, emphasis should be placed on pragmatic and cost‐effective interventions, potentially including maternal education on fetal movement awareness. Reduced fetal movements can precede fetal death in cases with abnormal umbilical cord characteristics with long‐lasting fetal decompensation. For example, reduced maternal perception of fetal movement is independently associated with nuchal cord and true cord knot before stillbirth.^58^ Although the evidence of the effectiveness of maternal education on fetal movement awareness in preventing stillbirth remains uncertain, it could be used as an inexpensive potential strategy to mitigate preventable stillbirths and narrow global disparities in perinatal outcomes.^61^

### 5.6 Future research directions and evidence gaps

Future efforts should focus on standardizing ultrasonographic definitions and developing validated biomarkers or functional indices to quantify umbilical cord compromise. Prospective multicenter registries could help clarify the natural history of different cord abnormalities and refine risk stratification. Advanced imaging and computational modeling might improve understanding of fetal hemodynamics and hypoxic progression in cord pathology. Despite the ethical constraints precluding randomized trials, well‐designed observational studies and international consensus on definitions are urgently needed. Until more robust evidence emerges, management might best be guided by a cautious approach, prioritizing surveillance tailored to maternal and fetal conditions and grounded in shared decision‐making principles.

For a summary of essential clinical points related to umbilical cord anomalies, see Box 1.

BOX 1 **Key points for clinicians**

- Umbilical cord anomalies constitute a recognized but largely unpredictable risk factor for adverse perinatal outcomes.
- Certain umbilical cord abnormalities are frequently observed in both liveborn and stillborn infants; histopathological placental analysis is critical to distinguish mere association from a presumptive causal relationship, revealing features such as fetal vascular malperfusion.
- Potential mechanisms of fetal death can include:
  ⚬ sudden interruption of umbilical blood flow due to obstructive compression or traction of umbilical vessels
  ⚬ placental hypoperfusion secondary to mechanical compression of cord vessels, potentially resulting in fetal growth restriction
  ⚬ fetal hemodynamic instability, which might progress to cardiac failure.
- Continuous surveillance cannot prevent abrupt cord occlusion but might facilitate early detection of progressive fetal compromise in selected high‐risk cases.
- Selective surveillance might confer benefit in defined clinical scenarios, although its overall efficacy remains limited.
- Standardized assessment protocols and the development of predictive biomarkers or tools are essential to plan strategies of reducing umbilical cord‐related preventable stillbirths.

## 6 CONCLUSION

Cord anomalies are a recognized risk factor for adverse perinatal outcomes but remain inherently difficult to prevent. Selective surveillance might be valuable in specific clinical scenarios, although its effectiveness is limited and must be balanced against the risk of over‐intervention. Standardization of ultrasonographic assessment and histopathological evaluations, together with the development of robust predictive biomarkers, are urgently needed to improve risk stratification and guide evidence‐based management, with the goal of reducing preventable stillbirth.

## AUTHOR CONTRIBUTIONS

LA conceived the manuscript. GB supervised. LA wrote the manuscript. CP, VM, and GB reviewed and revised the manuscript. All authors contributed to the manuscript revision; they have all read and approved the manuscript.

## CONFLICT OF INTEREST STATEMENT

The authors declare no conflict of interest.

## ETHICAL APPROVAL

Not applicable.

## CODE AVAILABILITY STATEMENT

Not applicable.

## CONSENT FOR PUBLICATION

Not applicable.

## Data Availability

Data sharing is not applicable to this article as no new data were created or analyzed in this study.
